# A Swept source optical coherence tomography angiography study: Imaging artifacts and comparison of non-perfusion areas with fluorescein angiography in diabetic macular edema

**DOI:** 10.1371/journal.pone.0249918

**Published:** 2021-04-08

**Authors:** Dominika Podkowinski, Sophie Beka, Anna-Sophie Mursch-Edlmayr, Rupert W. Strauss, Lukas Fischer, Matthias Bolz

**Affiliations:** 1 Department of Ophthalmology, Kepler University Clinic, Johannes Kepler University, Linz, Austria; 2 Johannes Kepler University, Linz, Austria; 3 Medical University Graz, Graz, Austria; 4 Software Competence Center Hagenberg GmbH (SCCH), Hagenberg, Austria; Massachusetts Eye & Ear Infirmary, Harvard Medical School, UNITED STATES

## Abstract

**Purpose:**

Swept Source Optical coherence tomography angiography (SS-OCTA) is a novel technique to visualize perfusion and vascular changes like ischemia in patients with diabetic retinopathy. The aim of this study was to compare non-perfusion areas on conventional fluorescein angiography (FA) with those on SS-OCTA using detailed manual annotation in patients with diabetic macular edema (DME) and to evaluate possible artifacts caused by DME on SS-OCTA.

**Methods:**

27 eyes of 21 patients with DME were analyzed in this prospective, cross-sectional study; on all, standard ophthalmological examination, SS-OCTA and FA imaging were performed. Early-phase FA and SS-OCTA images were analyzed for capillary dropout and foveal avascular zone (FAZ) was measured on both modalities. Artifacts in SS-OCTA imaging caused by DME were marked and analyzed.

**Results:**

The mean age of the patients was 62.6 ± 11.5 years. On FA the mean size of the annotated non-perfusion areas was 0.14 ± 0.31 mm^2^ whereas the mean size in SS-OCTA was 0.04 ± 0.13 mm^2^; areas marked on FA were statistically significantly larger than on SS-OCTA (p<0.01). Mean size of FAZs was similar between FA and OCTA images. (p = 0.91). Seven eyes (25.9 percent) showed imaging artifacts due to DME in SS-OCTA.

**Conclusion:**

SS-OCTA is a valid tool to analyze capillary perfusion status of patients with DME, although areas of non-perfusion were measured smaller than in conventional FA. More non-perfusion areas were found on SS-OCTA images. FAZ measurements were similar using the two modalities. However, SS-OCTA is prone to artifacts and therefore requires reviewing of imaging results: up to 25 percent of the analyzed eyes showed artifacts on OCTA, which occurred in the areas of diabetic macular edema and did not correspond to capillary drop out.

## Introduction

Diabetic retinopathy is one of the leading causes of visual loss in the working age population; especially diabetic macular edema (DME) [1]. DME, if left untreated, is a common cause of vision impairment and the prevalence of DME is rising continuously worldwide [2]. A recent pooled analysis of data showed an overall prevalence of 34.6 percent for any diabetic retinopathy (DR) and 6.81 percent (6.74–6.89) for DME worldwide; in total numbers there are approximately 93 million people with DR and 21 million with DME, making it a global health issue [3].

Fluorescein angiography (FA) is currently the gold-standard modality to examine the retinal microvasculature and to delineate areas of capillary non-perfusion in the retina. Analysis of the foveal avascular zone (FAZ) on FA images can investigate its effect on microvasculature and therefore be an indicator of severeness of DME [2]. The SAVE II protocol was recently developed and proposed as a new classification system for DME severity; it also comprises the enlargement of the FAZ to distinguish between different edema types [4, 5]. However, conventional FA has some disadvantages, especially being an invasive procedure including venipuncture, and also reports of anaphylaxis and even death related to dye injections have been documented [6]. Furthermore, imaging acquisition and analysis can be challenging due to compliance and reduced image quality by retinal hemorrhages. Another disadvantage of FA is that it delivers only two-dimensional images.

Recently, the novel modality of optical coherence tomography angiography (OCTA) as a non-invasive technique was introduced; it enables three-dimensional visualization of the choriocapillaris and retinal microvasculature by using detection of blood movement within the vessels. Basically, OCTA compares the decorrelation signal (differences in the OCT signal intensity or amplitude) between OCT B-scans taken at the same cross-section to further generate a map of blood flow [7, 8]. Swept source optical coherence tomography (SS-OCT) is working with higher wavelengths and applying ultrahigh speed; therefore, the visualization of deeper layers is further improved [9]. However, a “non-perfusion” signal on SS-OCTA can be caused by massively reduced blood flow instead of real non- perfusion. As every OCT system contains noise, thresholding was introduced to be able to interpret regions, highly impacted by noise. This necessity may additionally lead to suppressed visibility of moving erythrocytes and create areas of wrong non-perfusion [10].

So far comparisons of non-perfusion areas of the two modalities vary between studies. Different definitions were provided for these non-perfusion areas and studies did not solely focused on these areas and provided no detailed analysis of the non-perfusion areas [11, 12].

Hence the aim of this study was to compare areas of non-perfusion detected on FA images with those on SS-OCTA in detail, using manual annotations.

Furthermore, image artifacts of different origin in OCTA are common and a classification system of artifacts was previously established [13, 14]. As in FA imaging OCTA may be impacted by retinal hemorrhage too. However it has been reported, that analysis of retinal perfusion status can be made more precisely in areas with hemorrhage than on FA images in patients with retinal vein occlusion [15].

As OCTA is a rather novel imaging technology, artifacts caused by DME have been described in the literature, however reports of the prevalence of such artifacts are missing. Therefore we investigated how, and how often DME may impact SS-OCTA images in this study cohort and possibly produce a flow-negative area in the images, which may be misinterpreted as capillary non-perfusion.

## Material and methods

In this prospective, cross-sectional study we recruited 21 patients suffering from diabetic retinopathy with diabetic macular edema. Patients with both type I and type II diabetes were included at the Department of Ophthalmology at the Kepler University Clinic (Linz, Austria) between January and October 2018. This prospective study was approved by the Ethics Committee of the Federal State Upper Austria (approval number: B-142-17). Informed written consent was obtained after receiving explanation prior to enrollment. The study was conducted according to the Declaration of Helsinki. All patients underwent a full ophthalmological examination with slit lamp examination including dilated fundoscopy. The extent of diabetic retinopathy was determined based on Early Treatment Diabetic Retinopathy Study criteria [16]. All patients underwent SS-OCTA and FA imaging. SS-OCTA imaging was performed using the SS-OCT Plex Elite 9000 (Zeiss Medical, Dublin, CA; USA), operating at a scan speed of 100,000 A-scans per second with an axial resolution of 6.3 μm. SS-OCTA imaging was performed using a randomized order with 15 mm x 9 mm, 9 mm x 9 mm, 6 mm x 6 mm and 3 mm x 3 mm fields of view centered on the fovea. FA imaging was performed using Spectralis HRA+OCT (Heidelberg Engineering, Heidelberg; Germany). Early-phase images were captured at 30–45 seconds with a field of 30°.

Exclusion criteria were the presence of any severe media opacity, proliferative diabetic retinopathy (PDRP), history of panretinal laser photocoagulation (PRP) and all other retinal pathologies Further we excluded patients with a high refractive error over +/ -3diopters.

### Image analysis

Early-phase FA and SS-OCTA images were exported as TIFF files. All areas of capillary dropout and the FAZ on early-phase FA scans were analyzed and measured by one well-trained and masked investigator (D.P.) and independently reviewed by a second grader (S.B.). Manual annotation of capillary drop out on FA and SS-OCTA images was performed using Adobe Photoshop® software (Adobe Systems, Inc., San Jose, CA) Ischemic areas were analyzed qualitatively. In order to differentiate between true ischemic areas and dark areas, which may be caused by cotton wool spots or retinal hemorrhages, color fundus images were used to exclude those areas from measurements. In the next step, both investigators analyzed and measured all possible ischemic areas of the retina vasculature on 6 x 6 mm SS-OCTA scans, after review of the automatic segmentation of retinal layers and manual correction if necessary. In case of insufficient image quality, 9 x 9 mm or 15 x 9mm scans were used. For the analysis of ischemic areas the retina OCTA slab was used. Slabs are en face images obtained by projecting the flow information restricted to a particular depth within the OCTA volumetric acquisition, relative to a specific preset area The retina slab corresponds to the vasculature visible on FA images and demonstrates perfusion between the inner limiting membrane and 70μm above the retinal pigment epithelium.

Additionally, the investigators recorded areas on SS-OCTA images, which were graded as areas of artifacts: these were considered as zones, which appeared on OCTA as a gray, clearly defined area, which didn’t follow the capillary perfusion pattern. Adjudication was reached by review of examiners to reach consensus. Artifacts caused by blinking or small movement (white or black lines) were not considered as areas of interest for this analysis. Images were overlaid manually using vessel crossing as anatomical landmarks by the investigator (D.P.) after manual annotations.

Image analysis and quantification of both FA and SS-OCTA scans were performed automatically using the Image Processing Toolbox in Matlab 2017b (Matlab 2017b, The MathWorks Inc., Natick, Massachusetts; United States). Images were binarized and areas were calculated in pixels and gray values. Binarization was performed applying morphological operations, e.g. closing (imclose) followed by Otsu Thresholding (imbinarize) [17]. We converted the pixel into mm^2^ based on the resolution and image size of the analyzed images.

### Statistical analyses

All statistical analyses were performed using SPSS v 25.0 software (SPSS, Chicago, Illinois, USA). All of the analysed parameters were checked for normality (Shapiro–Wilk, p > 0.05; Q–Q plot). As normally distribution was given a Student’s t-test was used to test for statistical differences between the modalities. Normally distributed continuous variables are presented as mean±SD. Normally distributed continuous variables are presented as mean±SD. The formal significance level was set at p ≤ 0.05.

## Results

The mean age of the patients was 62.6 ± 11.5 years. Twelve patients were male and nine female. In total we included 30 eyes, and analyzed 27 eyes. Three eyes of two patients were excluded due to poor image quality. Two of the patients had diabetes mellitus type 1 and 19 patients presented with diabetes type 2. All patients had a history of anti-VEGF treatment. The shortest time interval from last treatment to imaging was four weeks. Ten eyes were treatment naïve; ten eyes had intravitreal injection a month prior to study inclusion. The last treatment for the remaining seven eyes was between 3 and 12 months.

**Fig 1:**
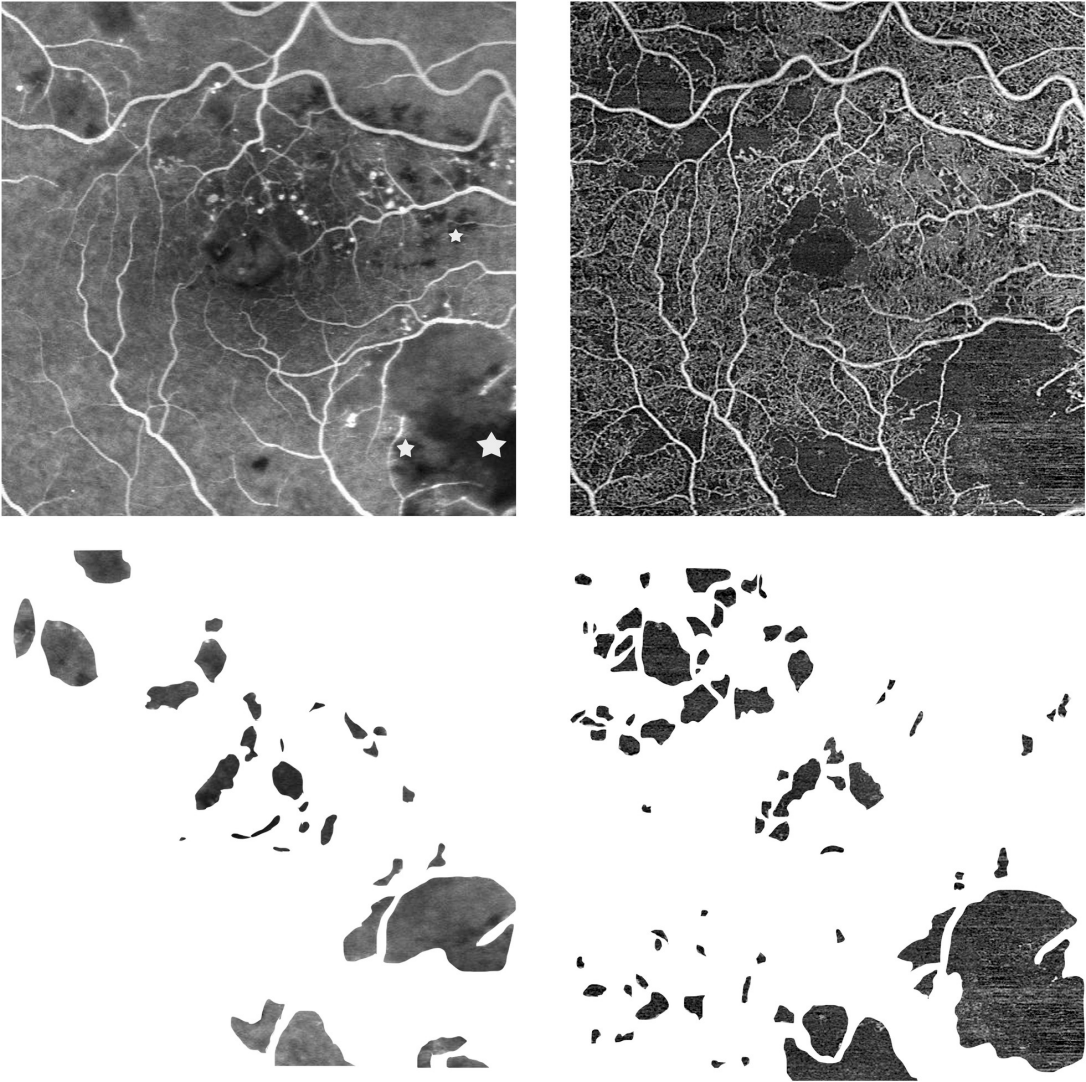
Example of areas of non-perfusion as determined by angiography fluorescein (FA) and Swept-Source Optical Coherence Tomography Angiography (SS-OCTA) images. Upper left: early phase FA image. Upper right: OCTA image of the retina slab. Lower left: Extracted annotated areas of non-perfusion from the FA image. Marked with white stars are areas which couldn’t be evaluated due to cotton wool spots and hyperreflective material. Lower right: Extracted annotated areas of non-perfusion from the OCTA image.

The mean area of FAZ on FA images was 0.30±0.23 mm^2^ and the calculated FAZ on SS-OCTA images was 0.26 ± 0.13 mm^2^. There was no statistically significant difference in FAZ between the two modalities. (p = 0.91). On FA images, 881 areas of capillary dropout were marked with a mean value of 939.35 ± 1,990.21 pixels, corresponding to 0.14 ± 0.31 mm^2^. The total number of identified avascular areas on OCTA was 1,429. The mean size of the areas on SS-OCTA was 797.08 ± 2,502.36 pixels (= corresponding to 0.04 ±0.13 mm^2^). The mean gray value of the ischemic areas on OCTA was 242.69 ± 14.18. An example of annotations is presented in Fig 1.

The mean size of the annotated ischemic areas was statistically significantly larger on FA images than on SS-OCTA images. (p<0.01) Except for two patients, there were more non-perfused areas identified on SS-OCTA images than on FA images.

### Quantitative analysis of artifacts on SS-OCTA

**Fig 2:**
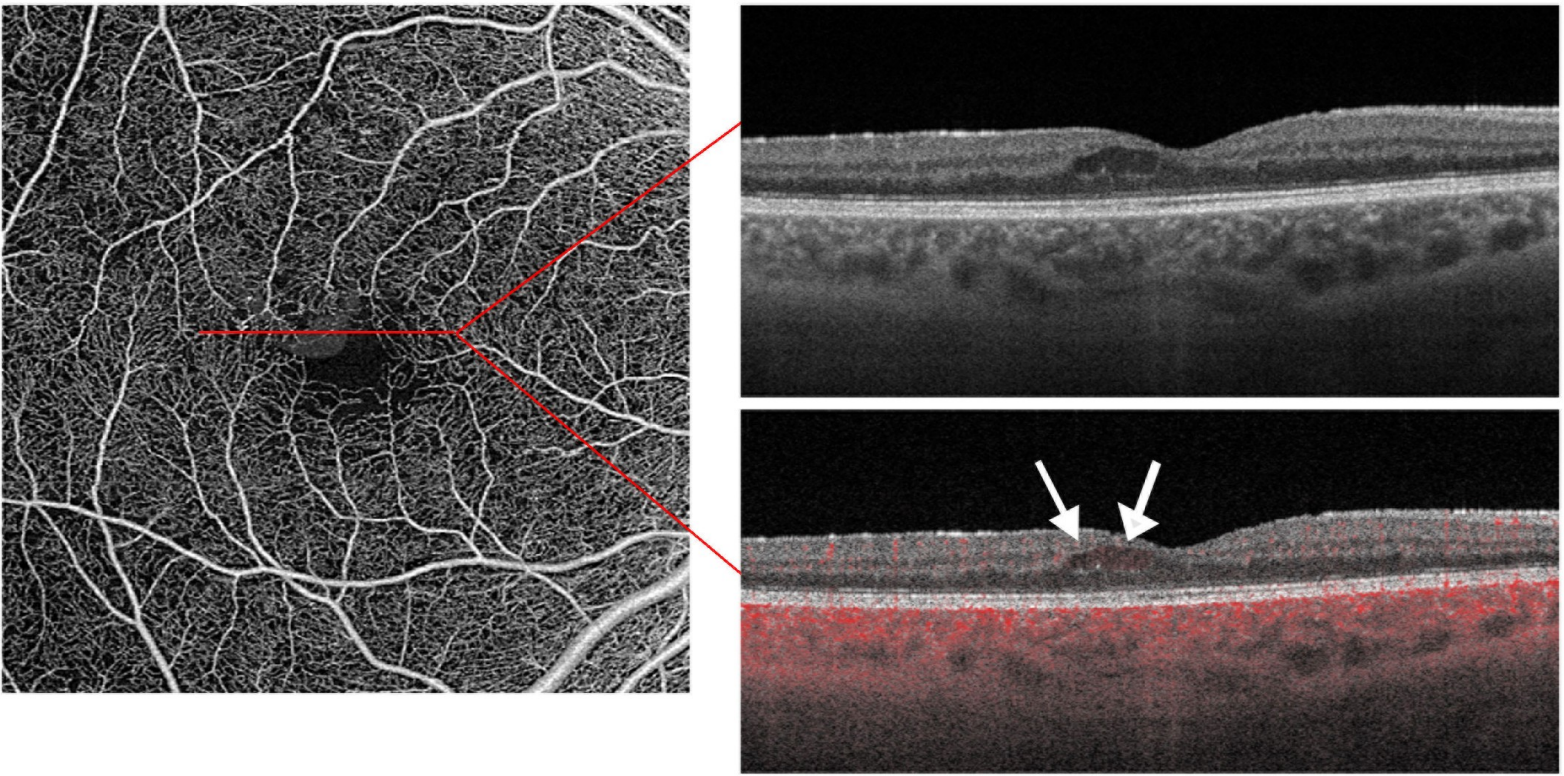
Example of annotated artifacts with flow in the cysts on OCTA images. On the left is an OCTA image of the retina slab. On the upper and lower right a B-scan of the location marked by the red line. On the upper right the B-scan is shown without perfusion representation, on the lower right with perfusion in red. The white arrows mark the perfusion visible in the cyst.

**Fig 3:**
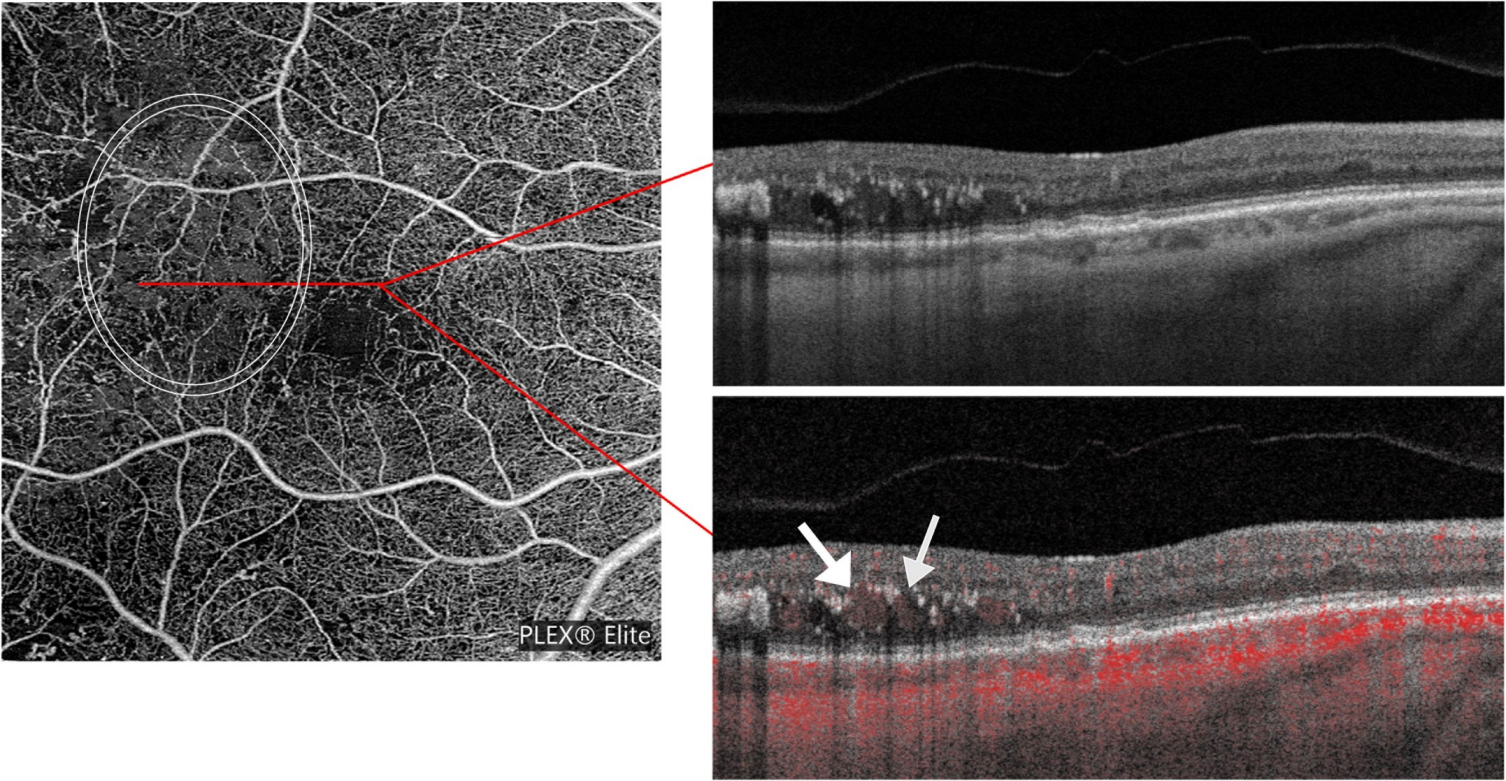
Example of annotated artifacts with flow around the cysts on OCTA images. On the left is an OCTA image of the retina slab. On the upper and lower right a B-scan of the location marked by the red line. On the upper right the B-scan is shown without perfusion representation, on the lower right with perfusion in red. The white arrows mark the perfusion around the cysts. Hyperreflective material is visible surrounding the cysts.

We detected areas to be considered as artifacts in seven eyes of seven patients (= 25.9 percent of the analyzed eyes). The mean area of graded artifacts was 5,296.02 ± 6,134.00 pixels (= 0.25 ± 0.29 mm^2^). In total, 24 areas were graded as artifacts. The artifacts had a mean gray value of 251.65 ± 1.92 pixels. Areas marked as artifacts had a larger mean area than areas of non-perfusion on SS-OCTA (p<0.01). We could identify two types of artifacts: one artifact presented as detected blood flow directly within intraretinal cysts. Two of seven eyes presented with this type of artifact. An example is displayed in Fig 2. The other five eyes showed flow around the cysts accompanied with hyperreflective material, without flow in the cyst themselves. Fig 3 demonstrates an example.

## Discussion

In this study we compared ischemic areas using detailed manual annotation and the FAZ as determined by conventional FA (which is still considered as gold-standard) with those in SS-OCTA imaging. The size of the FAZ was similar between FA and SS-OCTA imaging. Ischemic areas presented larger in size on FA than on SS-OCTA images. We found artifacts caused by diabetic macular edema in about 25% of the analyzed eyes in SS-OCTA images.

The FAZ measurements between FA and SS-OCTA were similar between the two image modalities. The FAZ is of special interest in diabetic retinopathy and diabetic macular edema: in the recently proposed SAVE II protocol, it is an essential part of grading to differentiate between different edema types and it furthermore also correlates with visual acuity [5]. SS-OCTA can be an additional, helpful tool for standardized grading protocols for DME. There are several studies that analyzed the FAZ in patients with diabetic retinopathy and diabetic macular edema and detected an enlarged FAZ on OCTA when compared to healthy controls [18, 19]. However it has to be noted, that FAZ measurements showed variability in healthy eyes [20]. Therefore analysis of the FAZ solely may be insufficient for the complex analysis of the perfusion status. Nevertheless, recent studies even showed that FAZ measurements could be of predictive value for progression of diabetic retinopathy [21]. Measuring the FAZ with OCTA could be a valid tool for the future, not only in patients with diabetic macular edema, but also in patients with macular edema due to retinal vein occlusion (RVO) [22].

Some studies compared macular perfusion using the Early Treatment Diabetic Retinopathy Study (ETDRS) classification in patients with diabetic retinopathy on FA and OCTA images [23, 24]. They used a SD-OCTA and demonstrated good agreement for the two modalities, whereas recently only a low agreement was detected in the comparison with a SS-OCTA [25]. However, this study didn’t analyze the areas of non-perfusion in detail and the classification based on ETDRS system may not be sufficient. Furthermore not all of the included patients presented with DME, which makes the group less homogenous and over half of the patients had prior PRP treatment due to PDR [25]. There is a controversial discussion to which extent PRP affects macular perfusion, but it may be a confounding factor analyzing perfusion [26, 27].

A novel study analyzing multiple imaging markers in patients with diabetic retinopathy found good comparison for non-perfusion areas between the two modalities, whereas another study showed more non-perfusion areas on SS-OCTA than on FA, which would be in accordance with our results [11, 12]. These discrepancies most likely originate in the different definitions of non-perfusion areas. Some studies provide a cut off for capillary dropout, such as *Cui et al*. where non-perfusion was defined larger or equal to a quarter of disc area. Compared to our study we didn’t analyze the periphery, meaning peripheral lesion could be missed. However the focus of our study was to analyze the exact size of non-perfusion areas for the two modalities, without a cut off. Due to the possible distortion of images in the periphery we focused on the central field of view to make the precise analysis of these regions possible. Furthermore not all FA and OCTA devices provide the possibility of wide-field imaging. Therefore our results are applicable in daily clinical practice, even without wide field imaging.

However, based on prior studies it is already known that SS-OCTA is a valid tool to detect non-perfusion areas in the periphery [28].

The strength of our study is the homogenous, clearly defined group of patients with DME and NPDR. Furthermore we analyzed not only the macular region for capillary dropout, but the complete imaged retina by taking advantage of the swept-source technology. Our analysis of ischemic areas beyond the FAZ reveals that the summarized areas delineated on FA are statistically significantly larger. The total number of identified areas, however, is higher in OCTA images. It may be speculated that this is based on the observed resolution of the different image modalities. FA requires a well-trained photographer to achieve high-quality images, especially beyond the posterior in case that ultra-wide field imaging cannot be applied, and as a consequence, minimal capillary dropout may not be identified as exactly as on OCTA images.

When analyzing OCTA images various causes of artifacts have to be considered. A recent study showed a good overview of possible imaging artifacts. The most common reasons for artifacts were projection and segmentation errors [29, 30]. As we used the retina slab for our analysis to achieve a good comparison with FA images projection artifacts are not affecting our analysis. However segmentation errors have to be considered when analyzing OCTA images, especially in DME as this often lead to segmentation errors. To avoid this type of artifact prior to image analysis we manually corrected the automated segmentation to ensure valid results.

Furthermore artifacts may originate from patient’s movement or blinking. These artifacts result in small black or white horizontal lines. We observed these artifacts in around 50 percent of our patients. Nevertheless these artifacts don’t impact the analysis of non-perfusion areas, as they are very subtle and can be easily attributed to movement or blinking based on their appearance [29].

Additionally, we found artifacts caused by diabetic macular edema in about 25 percent of eyes. A recent study published by *Farci R et al*. highlighted the relevance of differentiation between artifacts and real signals that originated from material in cysts [31]. The presented results suggest that the signal originates from floating particles of vascular origin of the parenchyma at septa of the cystoid spaces. Hence the gray signal on OCTA images should not be called artifacts, as it represents real flow. The study was conducted using the Optovue OCT-A device, which uses a different algorithm for flow analysis than the Plex Elite device. However we were able to identify these gray signals caused by cystoid spaces, too.

It can’t be excluded that the gray areas around diabetic macular edema are caused by stationary speckle noise. Materials which show a high reflectance of light produce interference and become then visible on OCTA images [13]. These extravascular findings on OCTA were further defined as “suspended scattering particles in motion” or SSPiM [32]. In this study the authors found a correlation of extravasated lipids as contributors to SSPiM and furthermore demonstrated the resolution of SSPiM with the formation of hard exsudates. However, it was also observed by other authors that these grey cysts didn’t necessarily resolve after formation of hard exsudates [31]. In our study we found two types of SSPiM as previously described by *Kashani et al*. One type was associated with hyperreflective material, but nevertheless about 30 percent of the artifacts did not show hyperreflective material. Therefore it is hypothesized that SSPiM is caused by a more severe blood retinal barrier breakdown; patients with diabetic macular edema presenting with SSPiM in OCTA demonstrated a poor structural response to treatment [33].

Another important factor when talking about OCTA analysis is the use of different devices. It was demonstrated that parameters derived from different devices are not necessarily equivalent due to different applied wavelengths and algorithms for perfusion detection [34]. In a recent study SSPiM were detected more frequently detected using the Plex Elite 9000 than the Optovue OCT-A [35]. Therefore the number of detected artifacts in our patient cohort may be higher if comparing it with other SS-OCTA devices.

The limitation of our study is the small sample size. Furthermore, we evaluated images only cross-sectionally and therefore the results could not investigate the change in capillary dropout over time or after treatment, for which varying results in respect of the FAZ and capillary perfusion were reported [36].

Although images were checked for segmentation errors, and corrected if needed, possible capillary analyzed “non-perfusion” on SS-OCTA may be still caused by artifacts, such as projection, segmentation, thresholding or other limiting factors using this imaging modality. As images of FA and SS-OCTA were overlaid using retinal vessels as biological landmarks, scaling errors of the two entities may have resulted in a possible mismatch between images.

In conclusion, SS-OCTA is a valid tool to analyze capillary perfusion status of patients with diabetic macular edema. We showed similar results for FAZ measurements between the two modalities. Non-perfusion areas were smaller on SS-OCTA images, but higher in number. The big advantage is its non-invasive operating mode, which can be of great value, especially in patients with diabetic nephropathy, when FA imaging may not be possible.

However, possible image artifacts must be taken into account when using and correctly analyzing OCTA images. In our homogenous study cohort of DME patients about 25 percent of the analyzed eyes showed artifacts on OCTA, which occurred in the areas of diabetic macular edema and did not correspond to capillary dropout. Further studies will be needed to analyze these artifacts over time and understand them better.
